# Acutely Decreased Thermoregulatory Energy Expenditure or Decreased Activity Energy Expenditure Both Acutely Reduce Food Intake in Mice

**DOI:** 10.1371/journal.pone.0041473

**Published:** 2012-08-22

**Authors:** Karl J. Kaiyala, Gregory J. Morton, Joshua P. Thaler, Thomas H. Meek, Tracy Tylee, Kayoko Ogimoto, Brent E. Wisse

**Affiliations:** 1 Department of Dentistry, University of Washington, Seattle, Washington, United States of America; 2 Department of Medicine, University of Washington, Seattle, Washington, United States of America; University of Santiago de Compostela School of Medicine - CIMUS, Spain

## Abstract

Despite the suggestion that reduced energy expenditure may be a key contributor to the obesity pandemic, few studies have tested whether acutely reduced energy expenditure is associated with a compensatory reduction in food intake. The homeostatic mechanisms that control food intake and energy expenditure remain controversial and are thought to act over days to weeks. We evaluated food intake in mice using two models of acutely decreased energy expenditure: 1) increasing ambient temperature to thermoneutrality in mice acclimated to standard laboratory temperature or 2) exercise cessation in mice accustomed to wheel running. Increasing ambient temperature (from 21°C to 28°C) rapidly decreased energy expenditure, demonstrating that thermoregulatory energy expenditure contributes to both light cycle (40±1%) and dark cycle energy expenditure (15±3%) at normal ambient temperature (21°C). Reducing thermoregulatory energy expenditure acutely decreased food intake primarily during the light cycle (65±7%), thus conflicting with the delayed compensation model, but did not alter spontaneous activity. Acute exercise cessation decreased energy expenditure only during the dark cycle (14±2% at 21°C; 21±4% at 28°C), while food intake was reduced during the dark cycle (0.9±0.1 g) in mice housed at 28°C, but during the light cycle (0.3±0.1 g) in mice housed at 21°C. Cumulatively, there was a strong correlation between the change in daily energy expenditure and the change in daily food intake (R^2^ = 0.51, p<0.01). We conclude that acutely decreased energy expenditure decreases food intake suggesting that energy intake is regulated by metabolic signals that respond rapidly and accurately to reduced energy expenditure.

## Introduction

Food intake and energy expenditure are linked by a complex biobehavioral control system that responds to the utilization and availability of metabolic energy in a process that favors energy homeostasis [1]–[3]. However, while homeostatic mechanisms defend robustly against states of energy depletion such as sustained fat loss [4], [5], the question of whether even fully intact control of food intake and energy expenditure can prevent incremental weight gain remains controversial [6], [7]. A number of clinical and pre-clinical studies have shown that weight gain caused by increased food intake is limited by increased energy expenditure [8]–[10]. Surprisingly, despite significant controversy regarding the role of ‘Western’ lifestyle-related decreased energy expenditure in contributing to the obesity pandemic [11], [12], relatively few studies have examined whether decreased energy expenditure actually causes reduced food intake so as to oppose weight gain. Accordingly, to evaluate the effect of acutely reduced energy expenditure on food intake, our study employed two strategies: 1) increased ambient temperature (T_a_) to reduce thermoregulatory energy expenditure (defined as energy expenditure for the primary purpose of maintaining body temperature) and 2) cessation of spontaneous exercise (locked running wheels) to reduce activity energy expenditure.

Processes regulating energy homeostasis are generally thought to balance food intake and energy expenditure over extended time periods (days) rather than more acutely (hours) [13] partly through mechanisms whereby changes in fat mass alter leptin and insulin signaling in multiple key brain regions [14]. However, rapid changes in both energy expenditure and food intake can occur to limit weight gain when the complementary variable is manipulated. Specifically, acute exposure of mice to a calorically-dense, highly-palatable diet increases food intake, but also increases energy expenditure within the first 24 h [15] and this increased energy expenditure is maintained if the diet is continued [8]. While multiple studies have shown that chronic housing at thermoneutrality decreases energy expenditure and reduces food intake [16], just one study has examined the effect of acutely raising ambient temperature (Ta) to 28°C on food intake, demonstrating reduced food intake during the first day of thermoneutrality [17].

The relationship between exercise and food intake is complex [7], [18]. Allowing mice access to running wheels increases energy expenditure and food intake in most, but not all [19], studies of spontaneous exercise and may be dependent on strain, diet and volume of exercise [18], [20]. These studies have not reported food intake within the first 24 h of exercise onset, and to our knowledge, no studies have examined the effect of acute cessation of exercise on food intake in mice. Thus, our study was designed to examine both the immediate effect of acutely reduced energy expenditure on food intake and to determine the initial accuracy of any compensatory food intake reduction.

In mice the thermoneutral range – the T_a_ at which thermoregulatory energy expenditure is minimized – lies between 27–31°C [21]. Thermoregulatory energy expenditure occurs by a variety of mechanisms (shivering and non-shivering heat production [22]), but can also involve heat generated by muscle contraction during exercise. At room temperature mice demonstrate high levels of spontaneous exercise by wheel running and high levels of ambulatory activity in their cage. Whether wheel running and ambulatory activity at 21°C are partly mediated by the need for thermoregulatory energy expenditure is unknown, and answering this question was a secondary goal of the current study.

## Materials and Methods

### Animals

Studies were conducted using male, adult (5–6 month) C57Bl/6J mice (Jackson Labs, Bar Harbor, ME). Prior to metabolic studies animals were housed individually in a temperature-controlled room (21±0.2°C) maintained on a 12 h light/dark cycle and were provided *ad libitum* access to water and pelleted rodent chow (Test Diet 5015, LabDiet Inc., Richmond, IN). All study protocols were approved by the Institutional Animal Care and Use Committee of the University of Washington, and were performed in accordance with the NIH Guide for the Care and Use of Laboratory Animals.

### Indirect Calorimetry

A standard 12 h light/dark cycle was maintained throughout the calorimetry studies. Prior to data collection, all animals were acclimated to home cages with running wheels; for the first 3 days the running wheels were locked, and during the subsequent 7 days the mice had free running wheel access. Mice were subsequently placed in metabolic cages located in the Animal Studies Core of the Nutrition Obesity Research Center (NORC) at the University of Washington. Energy expenditure measures were obtained using a computer controlled indirect calorimetry system (Promethion®, Sable Systems, Las Vegas, NV). The calorimetry system consists of 16 metabolic cages (identical to home cages with bedding) each equipped with water bottles and food hoppers connected to load cells for food and water intake monitoring, and all animals had *ad libitum* access to standard rodent chow and water throughout the study. All cages contained running wheels (4.5″ (11.5 cm) diameter, MiniMitter, Bend, OR) wired to record revolutions/second continuously using a magnetic reed switch, and all mice had been adapted to running as described above. Eight cages apiece are contained in two separate temperature- and humidity-controlled temperature cabinets (Caron Products & Services, Marietta, OH). The air within the cages is sampled through microperforated stainless steel sampling tubes located in the inner bottom rim of the cages to ensure that temperature inside the cages does not exceed the temperature within the chamber. Ambulatory activity and position are detected with XYZ beam arrays (BXYZ-R, Sable Systems, Las Vegas, NV) with a beam spacing of 0.25 cm. Respiratory gases are measured with an integrated fuel cell oxygen analyzer, spectrophotometric CO_2_ analyzer and capacitive water vapor partial pressure analyzer (GA3, Sable Systems. Las Vegas, NV). The system uses two GA-3 analyzers operating in parallel, devoted to eight cages apiece, to maximize throughput. Gas sensors are calibrated daily with 100% N_2_ as zero reference and with a span gas containing known concentrations of O2, CO2 with balance N_2_ (PraxAir, Tacoma WA). Promethion® utilizes a pull-mode, negative pressure system. Two multi-channel mass flow generators measure and control air flow (FR8, Sable Systems, Las Vegas, NV). The incurrent flow rate is set at 3000 mL/min. Water vapor is continuously measured and its dilution effect on O_2_ and CO_2_ are mathematically compensated for in the analysis stream [23]. Oxygen consumption and carbon dioxide (CO_2_) production are measured for each mouse at 10-min intervals for 1-min. Incurrent air reference values are determined after measuring every 4 cages. Respiratory quotient (RQ) is calculated as the ratio of CO_2_ production over O_2_ consumption. Energy expenditure is calculated using the Weir equation: Kcal/hr = 60*(0.003941*VO_2_+0.001106*VCO_2_) [24]. Ambulatory activity and wheel running were determined simultaneously with the collection of the calorimetry data. For ambulatory activity, consecutive adjacent infrared beam breaks in the y-axes, i.e. the length of the cage, are scored as an activity count, and a tally recorded every 10 min. Data acquisition and instrument control were coordinated by MetaScreen v. 1.6.2 and the obtained raw data was processed using ExpeData v. 1.4.3 (Sable Systems, Las Vegas, NV) using an analysis script detailing all aspects of data transformation. The script is available on request from the corresponding author.

### Body Composition Analysis

Body composition in terms of fat mass and lean body mass was evaluated in live, conscious animals in triplicate by quantitative nuclear magnetic resonance spectroscopy (EchoMRI™ 3-in-1 Animal Tissue Composition Analyzer; Echo Medical Systems, Houston, TX) [25], [26]. A system test is routinely performed at the beginning of each measurement day and the equipment is calibrated by scanning a calibration holder containing a known amount of fat to test the validity of measurement.

### Experimental Protocols (Figure 1)

**Figure 1:**
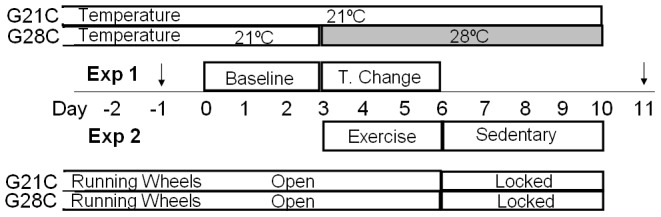
Study schematic. Initial temperature for both groups of mice (n = 8 mice/group) was 21°C. Starting on day -10 all mice were acclimatized to wheel running with the wheel locked for 3 days followed by 7 days of spontaneous running. Subsequently, continuous measurement of outcome variables was performed in metabolic cages for 10days (day 0–10). Experiment 1: Baseline measurement period (day 0–2) was equal in both groups of mice (n = 8) in that both groups were studied at 21°C. Ambient temperature was raised to 28°C for mice in the G28C group starting on day 3 and continued to the end of the study. Day 3–5 was evaluated as the temperature change (T. Change) period for Experiment 1. For Experiment 2 both groups had free running wheel access for spontaneous exercise. For both G21C and G28C day 3–5 was the exercise (EX) period evaluated. Running wheels were locked on day 6 and day 6–10 was evaluated as the sedentary (Sed) period for both groups. Throughout Experiment 2 ambient temperature was 21°C for G21C mice and 28°C for G28C mice. Arrows indicate the two days that body composition measurements were performed.

After the acclimation phase, baseline metabolic parameters were recorded for 3 consecutive days with both temperature cabinets maintained at 21.4±0.1°C and 41±0.2% relative humidity. Subsequently, the temperature in one of the cabinets (G28C, n = 8 mice) was gradually raised to 28.0±0.1°C and 41±0.2% relative humidity over a 4 h time interval at the end of the third baseline light cycle, while the other chamber was maintained at 21.4±0.1°C and 41±0.2% relative humidity. All metabolic parameters were continuously recorded for the subsequent 3 day period. At the end of the sixth light cycle, running wheels were electronically locked in all 16 cages and all metabolic parameters recorded for the next 4 days. Chamber temperature conditions during the sedentary (SED) period were kept the same as during the previous exercise (EX) period when spontaneous wheel running occurred (Figure 1). The three day exercise (EX) period which serves as the baseline period prior to exercise cessation is the same as the three day experimental period when T_a_ was changed to 28°C for the G28C group.

### Statistical Methods

All results are expressed as mean ± SEM. Light and dark cycle data for energy expenditure (kcal/h), food intake (g), ambulatory activity (beam breaks, bb) and wheel running (m) for each animal were averaged within each of the 3 experimental periods (days 1–3, 4–6 and 7–11 (except wheel running was blocked in the last period; see Figure 1)). Primary outcomes were energy expenditure and food intake, with the experimental design and statistical analysis tailored to these measures. Analysis of variance (ANOVA) tested for omnibus group-wide significance of energy expenditure and food intake; significant ANOVA results were followed by planned group comparisons using LSD between-subjects Student's t-tests. We also performed paired Student's t-tests for within-subjects comparisons involving the effects of temperature change and wheel running cessation vs. the appropriate comparison period (Figure 1). Only the relevant t-test comparisons are reported. Analyses involving group comparisons involving energy expenditure that were adjusted for continuous covariates were performed using the Univariate General Linear Model procedure in PASW Statistics (IBM, Somers, N.Y.). ANOVA was performed using Statistica (Version 7.1; StatSoft, Inc), Significance was established at p<0.05, two-tailed.

## Results

### Baseline Energy Expenditure, Wheel Running, Ambulatory Activity and Body Composition

Before beginning the metabolic cage study (Figure 1) the mice were divided into 2 groups (designated as G21C and G28C; n = 8/group) based on matching for total daily wheel running distance in the acclimation cages. Baseline evaluation within metabolic cages demonstrated that the two groups did not differ in dark cycle or light cycle food intake, energy expenditure, wheel running or ambulatory activity (Table 1, all p≥0.05). The groups exhibited minor, albeit significant, differences in body weight and lean body mass, both greater in group G28C, but both total and percent fat mass were equivalent between the two groups (Table 2). Thus, at baseline, the groups were well-matched on total energy expenditure, food intake and activity variables.

**Table 1:** Baseline EE characteristics for both groups of mice studied at 21°C.

	G21C		G28C	
per 12 h cycle period	DC	LC	DC	LC
mean EE (kcal/hr)	0.66±0.04	0.47±0.04	0.69±0.04	0.47±0.03
AA (beam breaks ×10^3^)	20.2±3.1	6.9±1.7	22.6±1.8	7.7±1.0
WR (km)	3.7±0.3	0.4±0.2	4.3±0.9	0.1±0.05
FI (grams)	4.3±0.2	1.2±0.2	4.6±0.3	1.3±0.1

All data mean ± SEM for n = 8 mice.

Mean EE (total energy expenditure), total WR (wheel running) over 12 hours, total AA (ambulatory activity) over 12 hours, total FI (food intake) over 12 hours, DC (dark cycle), LC (light cycle). Differences between Group 1 and Group 2 were not significant for any of the four variables by unpaired Student's t-test.

**Table 2:** Baseline body weight characteristics for both groups of mice studied at 21°C.

	G21C	G28C
Body weight (g)	27.5±0.4	29.7±0.5*
Lean body mass (g)	23.5±0.3	25.2±0.3*
Fat mass (g)	3.3±0.3	3.6±0.1
% Fat mass (%)	11.9±0.8	12.1±0.4

All data mean ± SEM for n = 8 mice.

Mean body weight, lean body mass, fat mass and percent fat mass shown for both groups at baseline (21°C T_a_). Differences between the two groups, G21C and G28C were significant for body weight and lean body mass by unpaired Student's t-test,

* p<0.05.

### Effect of Acutely Increased Ambient Temperature on Energy Expenditure

**Figure 2:**
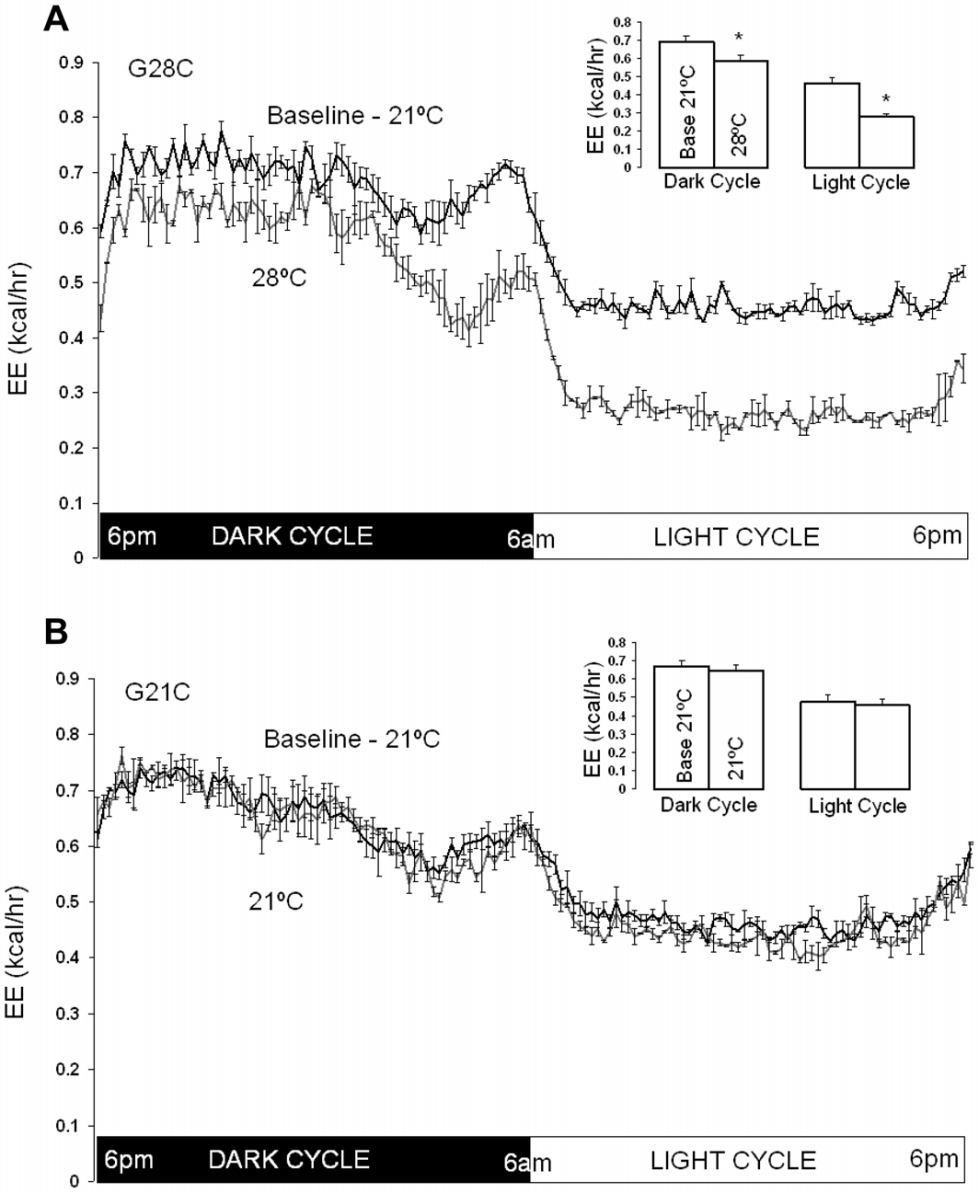
Effect of increased ambient temperature (T_a_) on total energy expenditure (EE) in G28C and G21C groups. A. G28C group (n = 8). Mean EE (kcal/hr) rate every 10 min during the entire photoperiod at baseline (21°C) and after increasing T_a_ to 28°C. Insert: Cumulative mean dark cycle and light cycle EE in G28C mice at baseline (Base 21°C) and after increasing T_a_ to 28°C (28°C). B. G21C group (n = 8). Mean EE (kcal/hr) rate every 10 min during the entire photoperiod at baseline (21°C) and after maintaining T_a_ at 21°C. Insert: Cumulative mean dark cycle and light cycle EE in G21C mice at baseline (Base 21°C) and after maintaining T_a_ at 21°C (21°C). All data shown as means ± SEM for 3 consecutive days, * signifies p<0.05 by pair-wise t-test.

As anticipated, raising T_a_ to 28°C, the lower limit of thermoneutrality in mice [21], decreased energy expenditure. Group G28C exhibited a mean reduction in light cycle energy expenditure of 40±1% (Figure 2A, p<0.001, by pair-wise comparison; group-wise comparison to G21C showed a 39±3% reduction, p<0.001). Comparison of mean energy expenditure during three consecutive days in each study period (Figure 2A), revealed that decreased energy expenditure was relatively uniform throughout the entire light cycle (Figure 2A) suggesting fairly constant thermoregulatory energy expenditure during this time period. Decreased energy expenditure occurred immediately during the first light cycle at thermoneutrality, and was consistent over all 3 consecutive light cycles at 28°C, as suggested by the small standard error bars in Figure 2A. Thermoneutrality also consistently decreased energy expenditure throughout the dark cycle, although the decrease was most evident during the last quarter of the photoperiod (Figure 2A). The mean energy expenditure decrease over the entire dark cycle for group G28C was 15±3% of baseline EE (Figure 2A, p<0.001, by pair-wise comparison). The pattern of decrease was again consistent over 3 consecutive recorded dark cycles (Figure 2A). As expected, comparing mean energy expenditure during three consecutive days in each study period in the G21C group showed that both light cycle and dark cycle energy expenditure remained unchanged (Figure 2B). Thus, changing T_a_ to thermoneutrality acutely reduces thermoregulatory energy expenditure. Thermoregulatory energy expenditure is a significant, consistent fraction of light cycle total energy expenditure at 21°C, but may be diminished during all but the last quarter of the dark cycle.

### Effect of Acutely Increased Ambient Temperature on Food Intake

**Figure 3:**
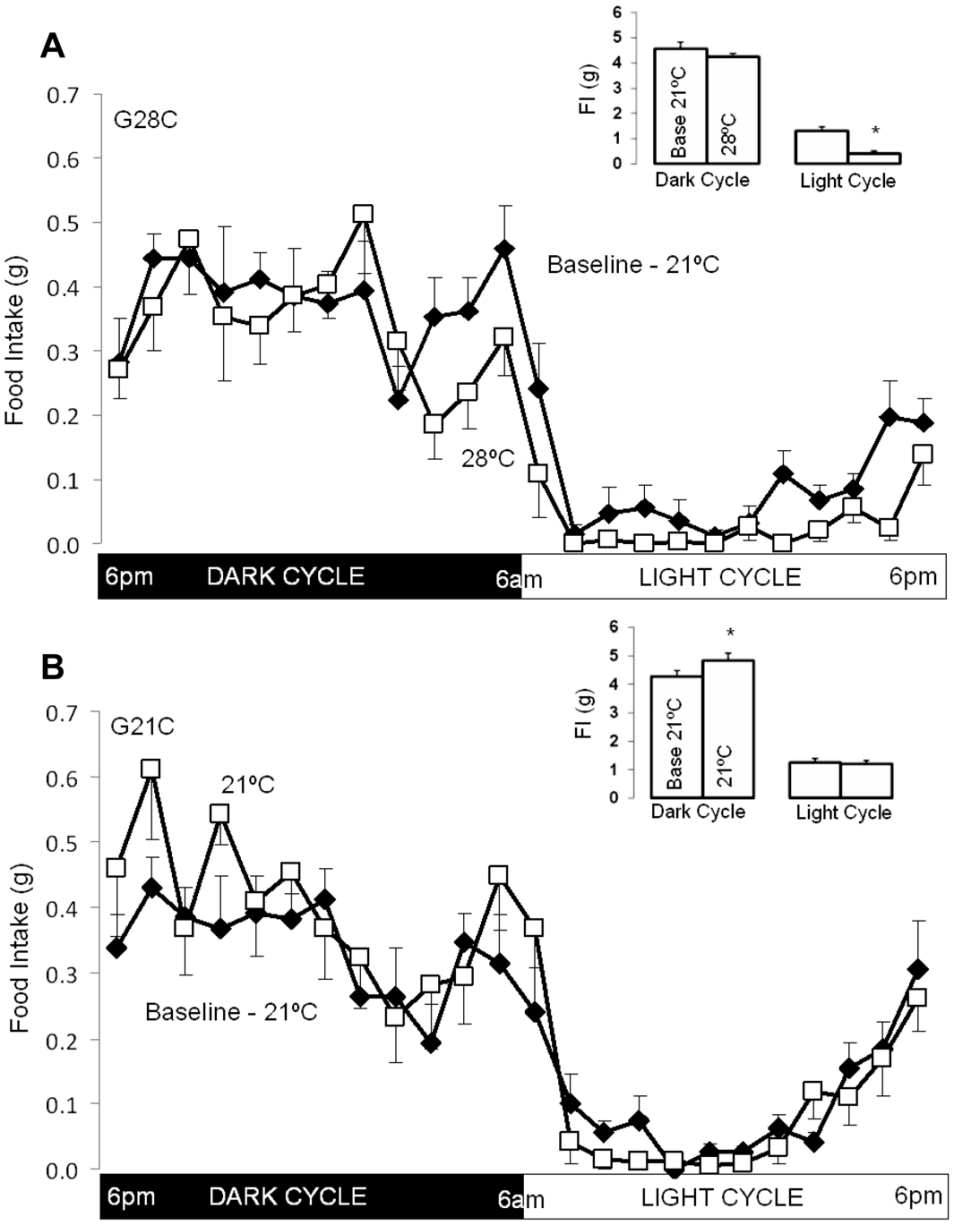
Effect of increased ambient temperature (T_a_) on food intake (FI) in G28C and G21C groups. A. G28C group (n = 8). Mean hourly FI (g) during the entire photoperiod at baseline (21°C) and after increasing T_a_ to 28°C. Insert: Cumulative mean dark cycle and light cycle FI in G28C mice at baseline (Base 21°C) and after increasing T_a_ to 28°C (28°C). B. G21C group (n = 8). Mean hourly FI (g) during the entire photoperiod at baseline (21°C) and after maintaining T_a_ at 21°C. Insert: Cumulative mean dark cycle and light cycle FI in G21C mice at baseline (Base 21°C) and after maintaining T_a_ at 21°C (21°C). All data shown as means ± SEM for 3 consecutive days, * signifies p<0.05 by pair-wise t-test.

Raising Ta to thermoneutrality in the G28C group decreased light cycle food intake by 65±7% (Figure 3A insert, p<0.001 by both pair-wise and group-wise comparisons) while light cycle food intake remained unchanged in G21C mice (Figure 3B, p = 0.5). The full expression of decreased food intake was reached immediately during the first light cycle after T_a_ was raised (0.36±0.11 g vs. 1.31±0.14 g at baseline), remaining unchanged during the subsequent two light cycles (0.43±0.15 g and 0.41±0.19 g, p = 0.7 and p = 0.8 vs. first light cycle; respectively) as indicated by the small error bars in Figure 3A. Comparison of mean hourly food intake during three consecutive days in each study period showed that reduced food intake occurred throughout the light cycle (Figure 3A). Total dark cycle food intake was unchanged in the G28C group (−0.3±0.3 g; p = 0.3, Figure 3A) while food intake increased slightly in group G21C (+0.5±0.1 g; p = 0.01, Figure 3B), though without any clear temporal pattern (Figure 3B), and the between group dark cycle food intake comparison was non-significant (p = 0.07). Surprisingly, as seen in Figure 3A, food intake was reduced during the final 3 h of the dark cycle in group G28C (0.7±0.1 g vs. 1.2±0.1 g; p = 0.005). Therefore, changing T_a_ to thermoneutrality acutely reduces food intake throughout the light cycle and during the last quarter of the dark cycle.

### Effect of Acutely Increased Ambient Temperature on Wheel Running and Ambulatory Activity

Raising Ta to thermoneutrality did not change dark cycle ambulatory activity (G21C: +1.2±3.1×10^3^ bb; p = 0.5 and G28C: +1.7±1.5×10^3^ bb; p = 0.3 by pair-wise comparison) or wheel running (G21C: +0.5±0.6 km; p = 0.4 and G28C: +0.6±0.4 km; p = 0.2 by pair-wise comparison) in either group. Similarly, no change was demonstrated in the low levels of light cycle ambulatory activity (G21C: +2.4±1.2×10^3^ bb; p = 0.1 and G28C: +0.4±1.2×10^3^ bb; p = 0.8 by pair-wise comparison). The low level of light cycle wheel running did not change in the G21C group (−0.07±0.7 km; p = 0.4 by pair-wise comparison) while a similarly small decrease in wheel running reached significance in G28C (−0.06±0.2 km; p = 0.04 by pair-wise comparison). Thus, acutely increasing T_a_ to thermoneutrality had minimal impact on spontaneous activity, suggesting that activity is not related to thermoregulation and is also not limited by ambient temperature in the thermoneutral range.

### Exercise Cessation Caused by Locking Running Wheels

As a second strategy to acutely decrease energy expenditure, running wheels were locked in both groups of mice (G21C and G28C) and all metabolic cage measurements recorded for the subsequent four days (Figure 1). For all outcomes the sedentary (SED) period was compared to the previous exercise (EX) period within groups (pair-wise analysis). Total wheel running was similar between groups prior to exercise cessation (G21C: 4.3±0.6 km and G28C: 4.9±1.0 km; p = 0.4). As discussed in the Methods the three day EX period is the same as the three day temperature change period for the first study.

### Effect of Acute Exercise Cessation on Energy Expenditure

**Figure 4:**
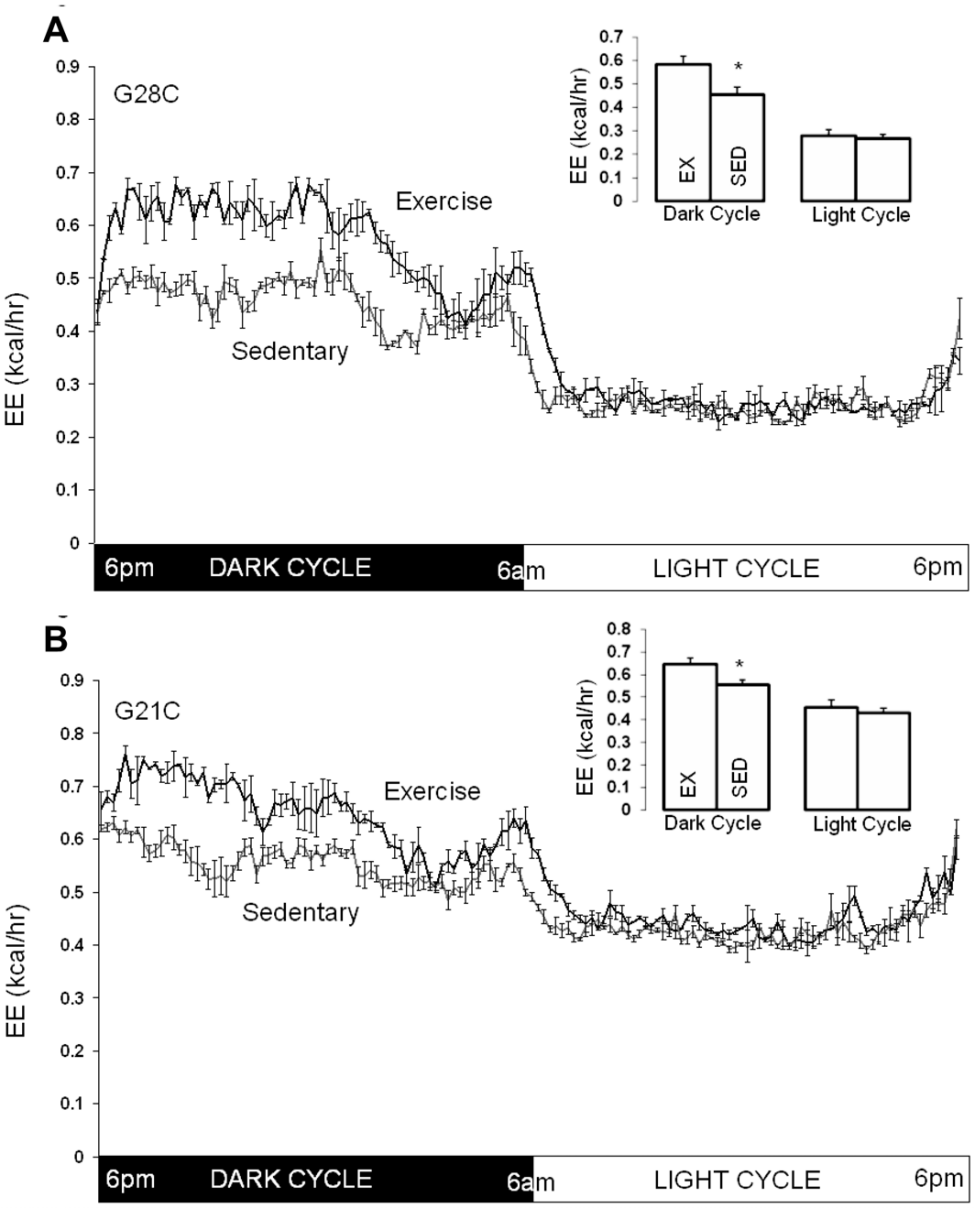
Effect of exercise cessation on total energy expenditure (EE) in G28C and G21C groups. A. G28C group (n = 8) housed at 28°C. Mean EE (kcal/hr) rate every 10 min during the entire photoperiod with Exercise and after running wheels were locked (Sedentary). Insert: Cumulative mean dark cycle and light cycle EE in G28C mice during the Exercise period (EX) and after running wheels were locked (SED). B. G21C group (n = 8) housed at 21°C. Mean EE (kcal/hr) rate every 10 min during the entire photoperiod with Exercise and after running wheels were locked (Sedentary). Insert: Cumulative mean dark cycle and light cycle EE in G21C mice during the Exercise period (EX) and after running wheels were locked (SED). All data shown as means ± SEM for 3(EX) or 4(SED) consecutive days, * signifies p<0.05 by pair-wise t-test.

Comparison of mean energy expenditure during consecutive days (EX three days, SED four days) in each study period demonstrates that exercise cessation acutely reduces dark cycle energy expenditure in both the G28C (−0.12±0.02 kcal/h, p<0.001, Figure 4A) and G21C groups (−0.09±0.02 kcal/hr, p<0.001, Figure 4B). Decreased energy expenditure occurred during the first dark cycle and remained consistently decreased across subsequent dark cycles. For both groups, decreased energy expenditure was most evident during the first half of the dark cycle (Figure 4A and B), the usual period of maximal wheel running, although this finding was more pronounced in group G28C (Figure 4A). As almost all running exercise occurs during the dark cycle, preventing wheel running had no effect on light cycle energy expenditure in G28C (Figure 4A) or G21C mice (Figure 4B). Thus, acute exercise cessation acutely decreases dark cycle energy expenditure consistent with a significant portion of dark cycle total energy expenditure attributed to activity energy expenditure.

### Effect of Acute Exercise Cessation on Food Intake

**Figure 5:**
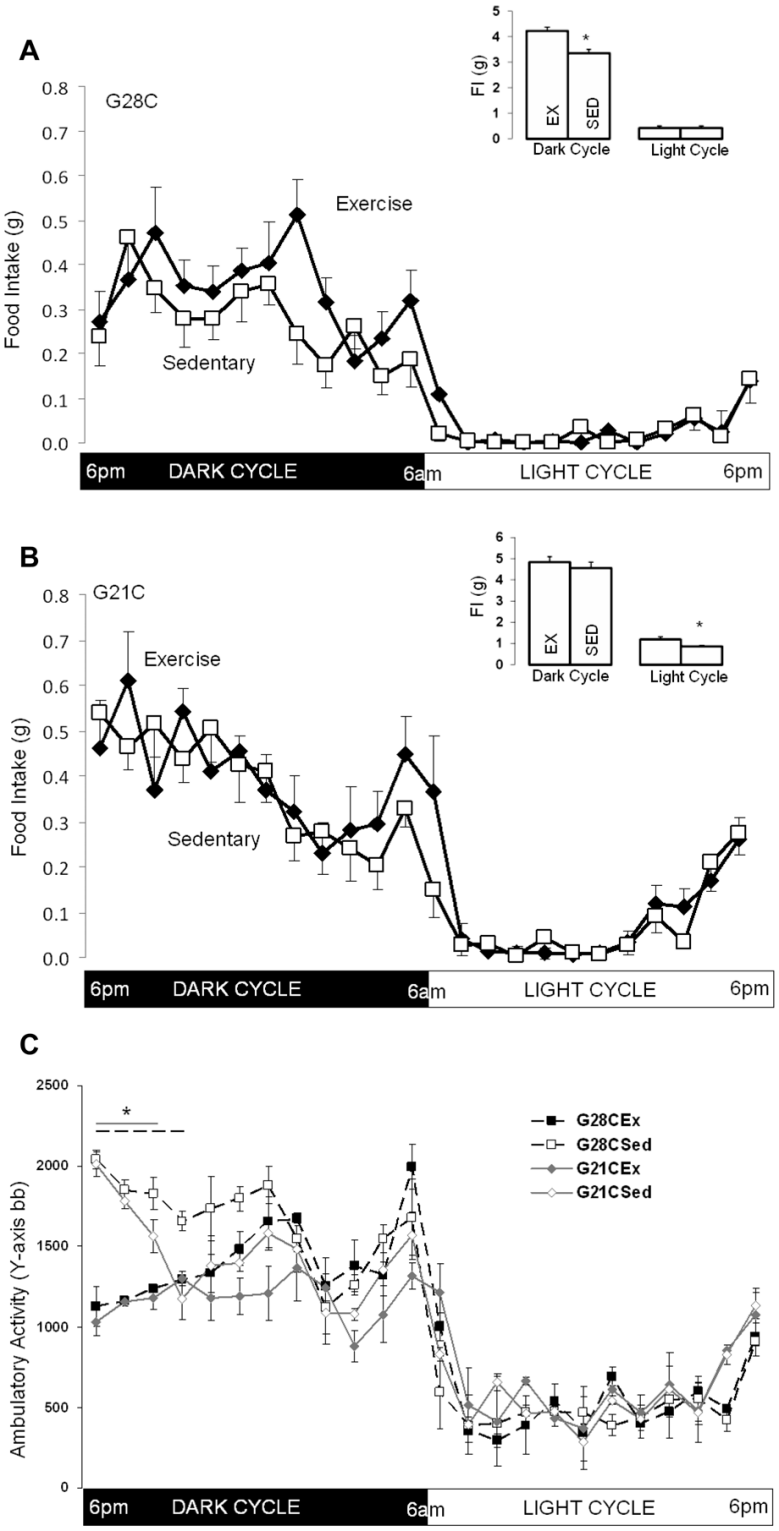
Effect of exercise cessation on food intake (FI) and ambulatory activity (AA) in G28C and G21C groups. A. G28C group (n = 8) housed at 28°C. Mean hourly FI (g) during the entire photoperiod with Exercise and after running wheels were locked (Sedentary). Insert: Cumulative mean dark cycle and light cycle FI in G28C mice during the Exercise period (EX) and after running wheels were locked (SED). B. G21C group (n = 8) housed at 21°C. Mean hourly FI (g) during the entire photoperiod with Exercise and after running wheels were locked (Sedentary). Insert: Cumulative mean dark cycle and light cycle FI in G21C mice during the Exercise period (EX) and after running wheels were locked (SED). C. Mean hourly ambulatory activity (Y-axis beam breaks) in both G28C and G21C groups (n = 8/group) during exercise period (Ex) and following exercise cessation (Sed). All data shown as means ± SEM for 3(EX) or 4(SED) consecutive days; for A and B * signifies p<0.05 by pair-wise t-test within groups, for C * signifies p<0.05 by one-way ANOVA for all hours indicated by the two lines (solid gray line G21C, dashed black line G28C).

At 28°C, exercise cessation reduced dark cycle food intake (−0.87±0.12 g, p<0.001, Figure 5A). Reduced food intake was evident already during the first dark cycle (3.4±0.3 g vs. 4.2±0.1 g during the exercise period) and was consistent over the subsequent two dark cycles (3.6±0.3 g and 3.3±0.3 g, p = 0.6 and p = 0.9 vs. first SED dark cycle; respectively). Comparison of mean hourly food intake during consecutive days (EX three days, SED four days) in each study period (Figure 5A) shows that food intake was reduced during most of the dark cycle. Light cycle food intake was not affected by exercise cessation at 28°C. In the G21C group dark cycle FI was not significantly reduced (−0.27±0.25 g, p = 0.3, Figure 5B) despite decreased energy expenditure. Surprisingly, light cycle food intake was slightly reduced in G21C mice (Figure 5B, EX: 1.2±0.1 g vs. SED: 0.85±0.1 g, p = 0.03), though the temporal pattern was not clearly consistent with a particular light cycle period being affected.

### Effect of Exercise Cessation on Ambulatory Activity

Total dark cycle ambulatory activity was not increased in either group of mice, G28C (+3.4±1.5×10^3^bb, p = 0.1) or G21C (+2.5±2.0×10^3^bb, p = 0.3). However, temporal ambulatory activity patterns demonstrated a consistent increase in activity during the first 3–4 hr of the dark cycle (Figure 5C) in both groups. Light cycle ambulatory activity was not altered by exercise cessation in either group, G28C (−0.8±1.0×10^3^bb, p = 0.8) or G21C (−0.5±1.7×10^3^bb, p = 0.5). Thus, acute exercise cessation appears to increase ambulatory activity during the early part of the dark cycle.

### Correlation Between Decreased Total Daily Energy Expenditure and Reduced Total Food Intake

For both groups we determined mean total daily energy expenditure (kcal/day) and mean total daily food intake (g/day) during each of the three study periods; 1) baseline, 2) temperature change with running exercise allowed, and 3) cessation of wheel running exercise. Raising T_a_ to thermoneutrality caused a 25±2% decrease in total daily energy expenditure and a 20±2% reduction in total daily food intake (both p<0.001 by pair-wise comparison). Cessation of exercise decreased daily energy expenditure by 16±3% and reduced total food intake by 18±1% (both p<0.001 by pair-wise comparison) in mice housed at 28°C, while mice housed at 21°C demonstrated a 10±1% decrease in daily energy expenditure (p<0.001) and a 9±4% reduction in total food intake, although the latter failed to reach significance (p = 0.07).

**Figure 6:**
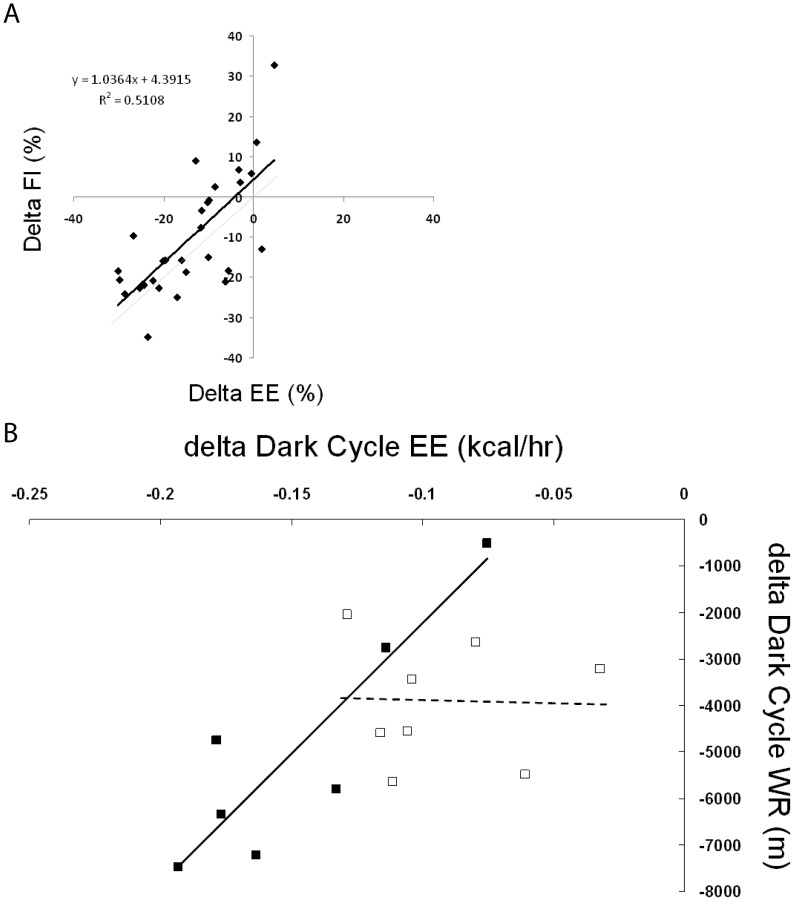
Correlation between change in energy expenditure (EE) and change in food intake (FI) or wheel running (WR) in individual mice. A Decrease in EE and FI for individual mice shown as % of value during the study period prior to the intervention (Baseline for Temp. change period (n = 15) and Exercise for the Sedentary period (n = 14). EE and FI data used were 3–4day averages for each distinct time period. Linear regression line (black) is parallel to line of identity (gray). B Decrease in dark cycle EE with exercise cessation vs. (negative) mean dark cycle WR distance during exercise period for mice housed at 21°C (open squares, dashed line) or 28°C (closed squares, solid line; note WR did not record for one mouse in the G28C group).

Subsequently, we determined the percent change in total daily energy expenditure and total daily food intake for individual mice. Percent change in total daily energy expenditure and percent change in total daily food intake were correlated for both experimental manipulations, 1) baseline to temperature change (n = 16 observations), and 2) exercise to exercise cessation (n = 16 observations). Automated food intake measurement failed in three instances yielding 29 (percent change energy expenditure, percent change food intake) coordinate pairs. As most animals generated two coordinates these 29 observations are not fully independent as is assumed in both standard bivariate Pearson regression and chi square analysis, and, accordingly, p-values for these tests were adjusted to reflect the loss of independence. The association between the percent change in daily energy expenditure and the percent change in total food intake was strong and significant (r = 0.71, p<0.01, Figure 6A). Notably, in 21 of the 29 observations, the percent reduction in food intake did not fully compensate for the energy expenditure decrease (p<0.01) such that food intake remained on average 3.9±1.9% higher than would have matched the decrease in energy expenditure (Figure 6A). Taken together, these data suggest that acutely decreased energy expenditure results in acutely reduced food intake, but that the food intake reduction may be systematically inadequate to match the energy expenditure deficit.

### Relationship Between Decreased EE Resulting from Exercise Cessation and Previous Amount of Running in Individual Mice

For individual mice we examined the change in dark cycle energy expenditure caused by exercise cessation in relation to the mean total dark cycle running activity during the exercise period. For mice housed at 28°C the correlation between decreased energy expenditure and decreased wheel running was high (Figure 6B, solid line, r = −0.89, p = 0.003) and the regression coefficient suggested that each km of running added ∼0.019 kcal/h to mean dark cycle energy expenditure. By contrast, mice at 21°C exhibited no correlation between the decrease in energy expenditure caused by exercise cessation and the prior amount of running (Figure 6B, dashed line, r = −0.02, p = 0.97), suggesting that decreased activity energy expenditure related to exercise cessation is being offset by an increase in another energy expenditure component.

We examined this concept further accounting for the fact that change-from-baseline values are generally correlated with the initial baseline values. First, we examined multiple regression models that estimated the change in energy expenditure (sedentary period-minus-exercise period) for both groups (G21C and G28C) with adjustment for 1) energy expenditure exhibited during the exercise period, 2) wheel running distance during the exercise period, and 3) the wheel running by temperature group interaction (as suggested by Figure 6B). The interaction term was not significant (p = 0.23) when the other variables were included in the model and was therefore excluded from the analysis. Table 3 shows the regression coefficients and interpretation of the final model. As predicted, energy expenditure during the exercise period was a significant determinant of the change in energy expenditure (higher energy expenditure values during the exercise period were associated with greater decreases in energy expenditure when wheels were locked; p = 0.005) and wheel running distance during the exercise period was also a significant determinant (greater wheel running distance during the exercise period was associated with larger decreases of energy expenditure when the wheels were locked; p = 0.023). Controlling for both variables at baseline showed that exercise cessation at 21°C decreased energy expenditure 0.053±0.016 kcal/h less than exercise cessation at 28°C (p = 0.005). This suggests that heat generated by wheel running at 21°C is used to support dark cycle core temperature homeostasis, estimated here as eliminating the need for, on average, 0.053 kcal/h of thermoregulatory energy expenditure, or ∼10% of total dark cycle energy expenditure.

**Table 3:** Multiple regression model for the adjusted effect of ambient temperature on the change in energy expenditure caused by locking running wheels during the sedentary period.

Parameter	Coefficient	SE	P	Partial r
Intercept	0.168	0.053	0.052	
Group = G21C	0.053	0.016	0.005	0.70
Group = G28C	reference			
EX Period EE	−0.337	0.098	0.005	−0.70
EX Period WR	−1.18 E-4	4.64 E-5	0.026	−0.59
Dependent variable is sedentary-minus-exercise period energy expenditure (kcal/hr)				

After statistically controlling for significant influences due to energy expenditure (EE) and wheel running (WR) in the exercise-allowed period (EX) prior to the sedentary period, energy expenditure in the group tested at 21°C decreased by 0.053±0.016 kcal/hr less than in the group tested at 28°C (p = 0.005). Note: the adjusted sedentary-minus-exercise decreases in energy expenditure had magnitudes of −0.073±0.01 kcal/hr for G21C and −0.126±0.01 kcal/hr for G28C.

### Reliability of Individual Differences in Energy Expenditure

We next evaluated the reliability of dark cycle and light cycle energy expenditure values in individual mice (Table 4). The within-subject reliability of energy expenditure from cycle to cycle is striking, suggesting that random factors do not contribute significantly to this outcome. Even following the change in T_a_ (G28C, period 1 to period 2), the correlations between individual energy expenditure between these periods were r = 0.89 (dark cycle) and r = 0.95 (light cycle). Indeed, correlations remained strong with the switch between period 2 and period 3 (exercise cessation) in both groups. Moreover, even the period 1 vs. period 3 correlations were all strong and significant. Given that body mass differed so little within or between groups (overall mean 28.6±1.59 g (SD), CV = 5.6%) body mass was a non-significant covariate (p = 0.9) in models that were examined to assess for possible effects of body mass on the between-period correlations. Thus, manipulations that change group energy expenditure do so in a way that largely preserve individual differences in this outcome.

**Table 4:** Reliability of within-subject energy expenditure values between study periods.

Energy Expenditure	P1 vs. P2	P2 vs. P3	P1 vs. P3
DC G21C	0.91(0.002)	0.90(0.003)	0.86(0.006)
DC G28C	0.89(0.003)	0.75(0.032)	0.80(0.018)
LC G21C	0.93(<0.001)	0.98(<0.001)	0.94(<0.001)
LC G28C	0.95(<0.001)	0.98(<0.001)	0.92(0.001)

The regression analysis data is shown as Pearson r (p-value) for all comparisons. Within subject analysis was performed for dark cycle (DC) and light cycle (LC) energy expenditure between the three study periods; period 1(P1) baseline, period 2 (P2) change in ambient temperature, and period 3 (P3) exercise cessation. Correction of regression analysis for body weight had no effect (p = 0.9) as coefficient of variation for body weight was only 5.6%.

### Effect of Reduced Energy Expenditure on Body Composition

**Figure 7:**
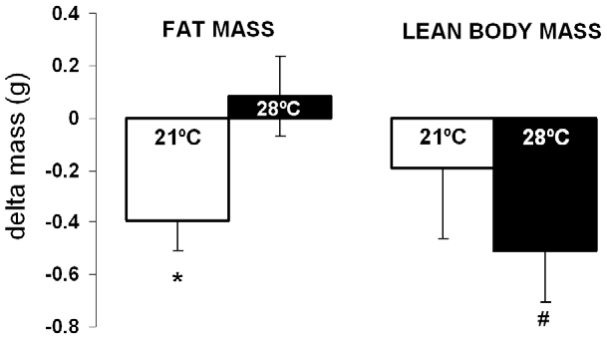
Change in body composition. Change in fat mass and lean body mass at the end of the study relative to baseline body composition. Mice housed at 21°C (white bars, n = 8) throughout the study are compared to mice housed at 28°C (black bars, n = 8) throughout all phases of the study. Data are shown as means ± SEM with * showing p<0.05 by both paired (within-group relative to baseline) and unpaired (between group) Student's t-test and # showing p<0.05 by paired Student's t-test.

Lean body mass and fat mass were measured by quantitative magnetic resonance at baseline and again eleven days later at the end of the study period (Figure 1) when both groups demonstrated decreased energy expenditure (decreased activity energy expenditure in mice housed at 21°C due to exercise cessation, and decreased activity energy expenditure and decreased thermoregulatory energy expenditure in mice housed at 28°C) as well as reduced food intake. Neither group demonstrated a change in body weight from the baseline evaluation (G21C: 27.5±0.4 g to 27.5±0.4 g and G28C: 29.7±0.5 g to 29.6±0.4 g). In mice housed at 28°C, fat mass did not change significantly (+0.1±0.2 g, p = 0.6 within group). Unexpectedly, the mice tested throughout at room temperature (21°C) lost a small amount of fat mass, relative to their baseline (−0.4±0.1 g, p<0.01), and also exhibited lower fat mass relative to the group tested at 28°C (G28C: 3.7±0.1 g vs. G21C: 2.9±0.1 g, p<0.001) (Figure 7). As lean body mass differed slightly at baseline, we evaluated this outcome only within groups. Lean body mass decreased (−0.5±0.2 g; p = 0.03) in mice tested at 28°C, but not in mice at 21°C (−0.2±0.2 g, p = 0.5)(Figure 7).

## Discussion

Contemporary models explaining the control of energy expenditure and food intake emphasize the complexity of the neuroendocrine control system that maintains energy homeostasis [2], [3], [14]. Importantly, whereas many studies demonstrate robust and effective homeostatic adaptations defending against the depletion of adipose tissue (e.g., [4]), the question of whether homeostatic mechanisms also can defend effectively against increasing fat mass remains controversial [3], [6]. This question is of vital importance given that decreased physical activity and abundant food characterize the modern environment, and given the need for effective therapies to prevent and treat obesity.

Existing studies have suggested that food intake is coupled to energy expenditure via mechanisms dependent on a longer-term integration of feedback related to energy storage, as demonstrated by the critical role for the adipocyte-derived hormone leptin in maintaining energy homeostasis [14], [27]. Our study demonstrates that acutely decreased energy expenditure resulting from increased T_a_ (decreased thermoregulatory energy expenditure) or exercise cessation (decreased activity energy expenditure) can cause acute and substantial reductions of food intake that persist for at least 3 days. The strong correlation between decreased energy expenditure and reduced food intake suggests the existence of a fast-acting control mechanism for energy balance limiting the potential for positive energy balance, and suggests that food intake is more readily adaptable to decreased energy expenditure than previously thought. However, our analysis also suggests that the reduction of food intake is inadequate – as indicated by the ∼4% systematic bias toward positive energy balance. This finding is important because a subtle but persistent error favoring positive energy balance could engender gradual weight gain provided sufficient time and food availability, consistent with the picture revealed by many animal and human studies. According to the model suggested by this view, weight gain over time does not indicate a lack of regulation as proposed by some investigators [28], rather it indicates a biobehavioral control system prone to instances of ‘steady state error’ [3].

While previous studies have shown that mice chronically housed at temperatures in the thermoneutral zone have significantly lower food intake than animals housed at standard room temperature [29], [30], to our knowledge only one study has shown that acutely increased T_a_ reduces food intake over a 24 h period [17]. Our study confirms and extends this finding by showing that the acute reduction in food intake is maintained over 3 days. Moreover, we found that reduced food intake primarily occurs during the light cycle, rather than during the dark cycle when the majority of food intake occurs. As the majority of energy savings due to reduced thermoregulatory energy expenditure also occurs during the light cycle, this finding suggests that light cycle food intake in mice housed at room temperature may be driven largely by increased thermoregulatory energy expenditure, as postulated more than 60 years ago by Brobeck [31], but to our knowledge largely untested until now. While previous studies suggest a complex relationship between exercise, food intake and weight gain [7], [18], we were unable to find previous work evaluating the effect of acute exercise cessation on food intake in mice or other rodent models. Our study demonstrated that exercise cessation acutely reduces energy expenditure and food intake at thermoneutrality. In addition, our data demonstrates a strong correlation between amount of exercise and decreased energy expenditure following exercise cessation in mice housed at 28°C. This may be relevant as one small clinical study demonstrated that imposed inactivity in moderately-active human subjects was not compensated for by reduced caloric intake over a two day period [32]. As our mice had high exercise levels (>4 km/dark cycle) it will be important to determine whether the mechanism underlying a rapid adaptation of food intake to energy expenditure in mice is relevant to humans engaged in typical levels of activity, or instead might be relevant only to subjects with high exercise levels.

While heat stress and increased core body temperature can cause reduced food intake [33], [34], a T_a_ of 28°C does not cause heat stress in mice [35], [36] and two recent studies in wild-type mice did not report significant core body temperature increase when T_a_ was raised to 30°C [37], [38]. Indeed, mice allowed to select their own T_a_ in a thermally graded alleyway typically prefer 28–31°C [39] Accordingly, heat stress or increased core body temperature are unlikely to explain reduced food intake in our study. Food intake increases energy expenditure, and this component of energy expenditure is often referred to as the thermic effect of food, defined as the energetic cost of digesting, metabolizing, transporting and storing ingested calories [40], [41]. However, studies evaluating thermic effect of food in mice consistently estimate that this component accounts for ∼5–8% of energy expenditure [18]. Thus a 30% reduction in food intake (the upper range of reduced food intake in our study) would be expected to cause only a 1–2% decrease in energy expenditure, i.e., much smaller than that observed. This finding suggests that our interventions (increased T_a_ and exercise cessation) did not decrease energy expenditure as a consequence of reducing food intake, but rather that food intake was decreased as a consequence of reduced energy expenditure.

Our study suggests the existence of, but does not identify, a mechanism that might rapidly reduce food intake in response to acutely decreased energy expenditure, and future studies are needed to pursue this goal. Reduced food intake is most commonly ascribed to physiological catabolic pathways (leptin/POMC/αMSH, CRH, nesfatin-1, CART, MCH, etc.) or pathological inflammatory mediators (TNFα, IL1β, etc.). The acute regulation of food intake in this study seems less compatible with a classic mechanism dependent on increased fat storage and/or increased circulating leptin, and previous studies have demonstrated that chronically-reduced food intake in mice housed at thermoneutral temperatures is not dependent on leptin [42]. To our knowledge, no studies have described changes in other catabolic neuromediators or inflammatory cytokines related to either acutely increased T_a_ or acute exercise cessation.

Recent comprehensive reviews have outlined the potential confounding effects of thermoregulatory energy expenditure that occur when studying energy homeostasis in rodents housed below the thermoneutral zone – stressing that genetic or pharmacological intervention to promote or prevent dietary weight gain could be altering thermoregulatory energy expenditure rather than more clinically-relevant pathways [21], [43], [44]. Our study emphasizes that thermoregulatory energy expenditure indeed is a significant component of total energy expenditure in mice housed at normal laboratory temperature and further demonstrates that thermoregulatory energy expenditure is a significant component not just of light cycle energy expenditure, but also of dark cycle energy expenditure even in mice that demonstrate substantial wheel running and ambulatory activity.

While ambulatory activity and wheel running are behaviors subject to biobehavioral modulation, the specific mechanisms controlling ambulatory activity and wheel running remain poorly-understood [45], [46]. However, a growing body of work in rodents demonstrates that ambulatory activity and wheel running may be altered in accordance with signals related to energy homeostasis. Specifically, hormonal mediators such as leptin and ghrelin, and neurohormonal factors such as melanocortins, Bsx, BDNF and serotonin, when genetically or pharmacologically manipulated result in altered spontaneous physical activity and energy balance [47]–[56]. Our study adds two novel findings to this emerging area. Firstly, increased T_a_, or the resulting reduced thermoregulatory energy expenditure does not acutely reduce wheel running. This suggests 1) that a warm environment *per se* does not reduce voluntary exercise in mice, and 2) that mild cold stress associated with normal laboratory temperature does not promote wheel running as a means for adaptive heat production. Importantly, our study suggests that wheel running at 21°C reduces the need for thermoregulatory EE during the dark cycle when wheel running is highest, potentially explaining why exercise cessation at 21°C was associated with lesser reductions in energy expenditure and food intake than at 28°C. An increase in thermoregulatory energy expenditure (and corresponding increased food intake) to compensate for reduced exercise-induced heat production could decrease the impact of decreased activity energy expenditure to reduce total energy expenditure and food intake. Together, our findings suggest that exercise does serve as a useful mechanism to generate heat in mice housed at standard laboratory temperature, as has been previously suggested for ambulatory activity in rats [57]. Nonetheless, the mice predominantly use thermoregulatory energy expenditure (presumably through shivering or stimulating brown adipose tissue) to maintain body temperature when faced with mild cold stress rather than increasing physical activity.

Secondly, our study suggests that ambulatory activity and wheel running are largely independently-controlled activity variables in wild-type mice as cessation of wheel running exercise did not change total ambulatory activity. Previous studies have been unable to fully resolve this question [18], but few studies have measured wheel running and ambulatory activity simultaneously over prolonged periods. Importantly, some interdependence between wheel running and ambulatory activity may exist as exercise cessation increased ambulatory activity during the first 3–4 h of each dark cycle suggesting a potential shared mechanism to increase physical activity during this specific time period. While ambulatory activity in mice may increase due to anxiety/novelty/change or merely because the time previously spent wheel running must now be spent doing something else [58], we submit that such mechanisms would be less likely to occur consistently during the same 3–4 h time period over four consecutive dark cycles. Importantly, beam break activity measures are an imprecise measure of true ambulatory activity and further studies are needed to accurately determine the effect of exercise cessation on ambulatory distance and speed.

In conclusion, we report that acutely reduced thermoregulatory energy expenditure and acutely reduced activity energy expenditure are associated with immediate and sustained (3 days) reductions in food intake. This suggests that as yet unknown mechanisms exist to reduce positive energy balance in the setting of acutely reduced energy expenditure. These findings may prove important to goals of identifying novel mechanisms to reduce food intake in working towards better treatments for obesity.
